# Clinical results of a lamina with spinous process and an iliac graft as bone grafts in the surgical treatment of single-segment lumbar pyogenic spondylodiscitis: a retrospective cohort study

**DOI:** 10.1186/s12893-022-01506-1

**Published:** 2022-02-11

**Authors:** Ke Tang, Weiyang Zhong, Xiaolin Wang, Xiaoji Luo, Zhengxue Quan

**Affiliations:** 1 Department of Orthopedic Surgery, The First Affiliated Hospital of Chongqing Medical University, Chongqing, China; 2 Department of Orthopedic Surgery, People’s Hospital of Yubei District, Chongqing, China

**Keywords:** Bone graft, Lumbar pyogenic discitis, Spinous process, Lamina, Iliac graft

## Abstract

**Background:**

A retrospective study compared the results of a lamina with spinous process (LSP) and an iliac graft (IG) as bone grafts in single-segment lumbar pyogenic spondylodiscitis (LPS) through one-stage-posterior-only approach with radical debridement and instrumentation.

**Methods:**

A LSP was placed in 17 patients (group A), and an IG was implemented in 20 patients (group B). The surgery time, surgery hemorrhage, hospital stay, drainage, and follow-up (FU) were recorded and compared. The erythrocyte sedimentation rate (ESR), C-reactive protein (CRP) level, visual analogue scale (VAS), Oswestry Disability Index (ODI), segmental angle, intervertebral height and bony fusion time were compared preoperatively and at the final FU.

**Results:**

All patients were followed-up for a mean of 27.94 ± 2.35 months in group A and 30.29 ± 1.89 months in group B, without a difference. The mean age was younger in group A than in group B (P < 0.05). The surgery time, surgery hemorrhage, and hospitalization cost were lower in group A than in group B (P < 0.05), except for the hospital stay and drainage time. 10 patients in group A had fever and 12 patients in group B. The ESR, CRP level, VAS and ODI scores were significantly decreased, and no significant differences were found between the groups at the final FU. The distribution of bacterial agents in blood culture was 1 case of *Aerobacter cloacae*, 2 of *Staphylococcus aureus*, 2 of *Escherichia coli*, and 1 of *Streptococcus viridis* in group A and 1 of *S. aureus*, 1 of *Staphylococcus warneri* and 2 of *Klebsiella pneumoniae* in group B. Pyogenic infection was observed in the pathological findings of all patients. No significant difference was found in the mean segmental angle or mean intervertebral height preoperation and at the final FU.

**Conclusion:**

The use of LSP could be an effective bone grafting for surgical management for the LPS while surgery is proposed as a good management strategy for single-segment LPS in carefully selected patients.

## Background

Lumbar pyogenic spondylodiscitis (LPS) is difficult to diagnose because of its insidious start and indolent course, and it is a rare infection with an increase in the growing number of human immunodeficiency virus (HIV) coinfections, bacterial resistance and population migration [1–3]. The diagnosis of LPS is usually delayed a few months and could be misdiagnosed and mishandled as a degenerative disease [3, 4]. LPS is often monomicrobial and most commonly due to *Staphylococcus aureus*. The management is mainly based on the correct results of culture and in vitro chemosensitivity assay [5, 6].

Most patients are cured after 6 weeks or more of antimicrobial therapy combined with lumbar brace, but a few cases may require surgical debridement and/or spinal reconstruction during or after antimicrobial therapy [5, 6]. LPS can result in destructive lesions or neurological impairment, which is indicated for surgery. Although the surgical approaches for LPS are controversial, surgical treatment can provide better pain relief and quality of life. After meticulous and radical debridement, bone grafts play a key role in surgery by curing LPS, as they can rebuild spinal stability and maintain alignment when patients suffering from neurological deficits are indicated for surgery [5, 7–9]. In our study, we compared a lamina with spinous process (LSP) and an iliac graft (IG) as bone grafts in treating single-segment LPS through a posterior-only approach with debridement and internal instrumentation.

## Methods

### Patient selection

From January 2014 to December 2016, 37 patients with single-level LPS were reviewed retrospectively and were divided into two groups. When performing a good communication with patients before surgical management, the strengths and weaknesses of the therapy plans using the two types of interbody fusion were completely explained. The anatomy, surgical technique and postoperative complications were introduced in detail so that the patients could think enough and choose the right way. The inclusion criteria were as follows: adult single-level LPS, a one-stage-posterior-only approach, instrumentation and interbody fusion, and patients indicated for surgery due to failure to respond to antimicrobial treatment, neurological deficits, or bone destruction affecting stability. The exclusion criteria were as follows: spinal tuberculosis, fractures, spine metastasis and cancer.

### Surgical procedure

**Fig. 1:**
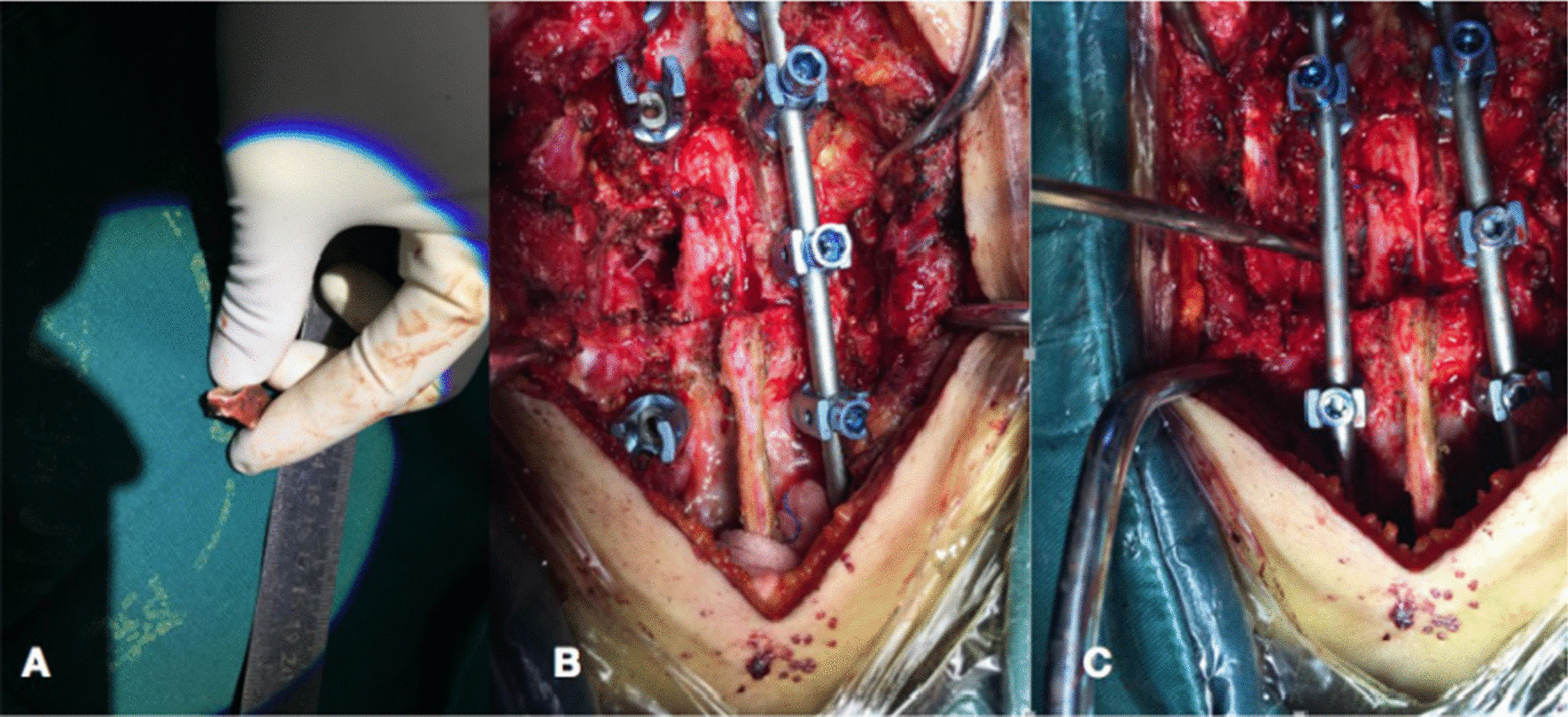
Photographs of one LSP that was implanted (**A**, **B**), and the LSP was verified for stability (**C**)

After the successful general anaesthesia and the patients placed in the prone position, through a midline incision, the posterior spinal elements, including the lamina and facet joints were fully exposed. The pedicle screws were fixed exactly and the decompression, complete debridement were performed. The LSP(Group A) or IG(Group B) was cut off for complete resection, and they were trimmed for a suitable bone graft (Fig. 1). According to the area remaining after complete debridement, one graft was implanted and locked with strong instrumentation. Vancomycin (1.0 g) mixed with gelatine was used locally around the graft. The drainage and lavage with saline were applied postoperatively. The specimens were sent for bacterial culture and pathological testing. The patients wore a brace for 6–8 weeks after surgery.

### Postoperative care

Rehabilitation therapist-guided ambulation exercises were started 1 week after the operation. All the patients were recommended to undergo antimicrobial therapy for 8–12 weeks after surgery. All patients underwent clinical and imaging examinations 1 week, 12-week, 6-month, 1-year and annually after surgery.

### Follow-up index

The FU data were recorded perioperatively and during FU. (1) The surgery time, surgery hemorrhage, hospital stay, drainage, the FU time and bony fusion time were recorded. (2) Pathological findings: tissue edema or inflammatory cell infiltration. (3) The segmental angle was recorded according to the Cobb method. (4) The intervertebral height was defined as the vertical height between the upper and lower vertebral bodies of the fused segment on lateral X-ray. (5) The VAS and ODI were recorded. (6) The ESR and CRP were recorded. Bony fusion was evaluated by X-ray and CT when necessary, by Bridwell criteria [10]. All radiographic data were reviewed and compared by one senior spine surgeon and one senior radiologist.

### Statistical analysis

The statistical analysis was performed using the Statistical Analysis System (SAS Institute Inc., Cary, NC, USA). The results are expressed as the mean ± SD. Differences with P values < 0.05 were considered statistically significant.

## Results

### Clinical assessments

**Fig. 2:**
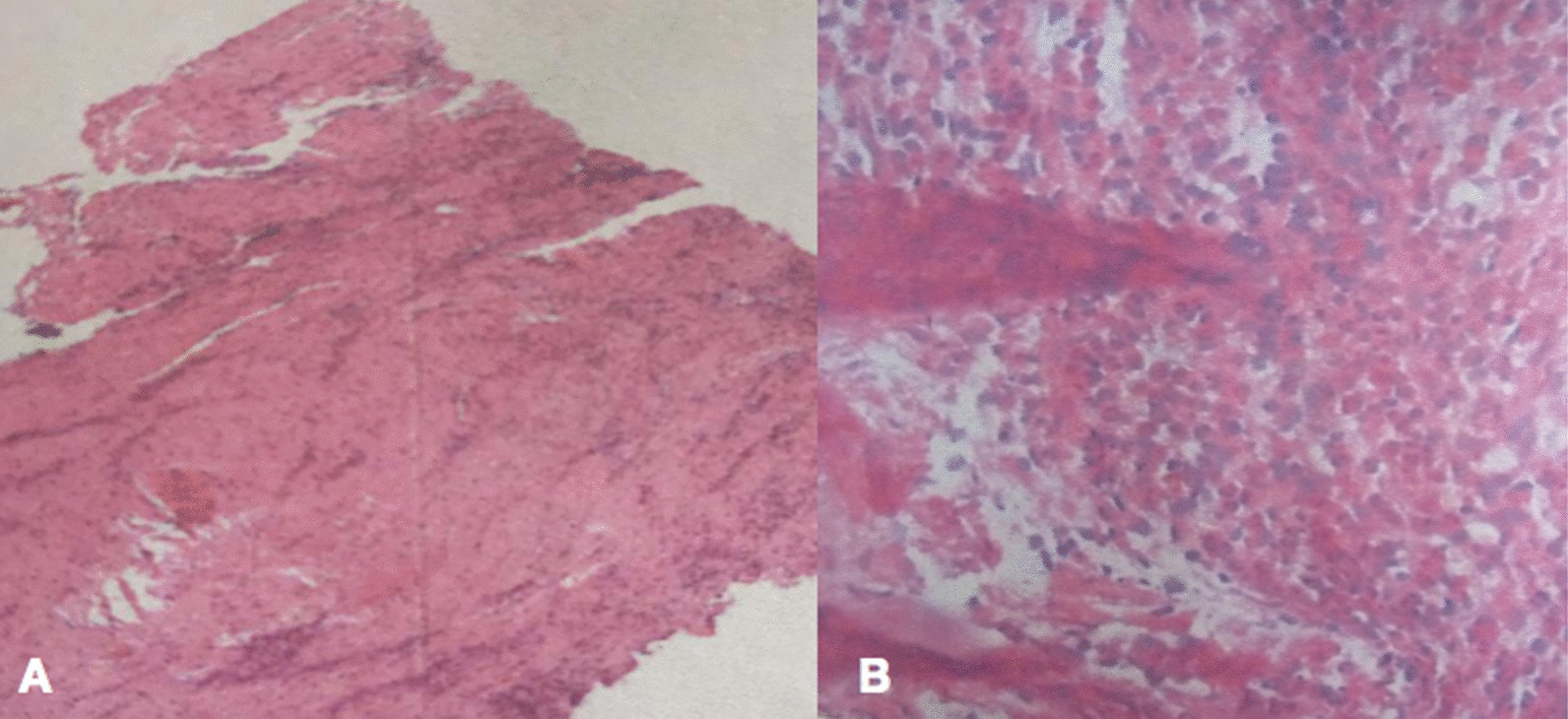
**A** The pathological findings in a patient aged 40–50 years old with lumbar pyogenic discitis (L4–5) **B** The pathological findings in a patient aged 30–40 years old with lumbar pyogenic discitis (L4–5)

All patients were followed up on average of 27.94 ± 2.35 months in group A and 30.29 ± 1.89 months in group B, with no difference (P > 0.05).The mean age was younger in group A than in group B (P < 0.05).The surgery time, blood loss, and hospitalization cost were lower in group A than in group B (P < 0.05), except for the hospital stay and drainage time (Table 1). 10 patients in group A had fever and 12 patients in group B without a difference (P > 0.05). The mean time of antibiotic therapy before surgery was 17.62 ± 3.76 days in group A and 13.86 ± 4.71 days in group B, without a difference (P > 0.05). The ESR, CRP level, and VAS and ODI scores were significantly decreased, no significant differences were found at immediately postoperation and at the final FU (Table 2). The distribution of bacterial agents in blood culture was 1 case of *Aerobacter cloacae*, 2 cases of *Staphylococcus aureus*, 2 cases of *Escherichia coli*, and 1 case of *Streptococcus viridis* in group A and 1 case of *S. aureus*, 1 case of *Staphylococcus warneri* and 2 cases of *Klebsiella pneumoniae* in group B (Table 3). In stool, urine or surgical material culture, no bacterium was isolated. Pyogenic infection was observed in the pathological findings of all the patients (Fig. 2).

**Table 1:** Information of the patients

	Group A	Group B	P value
No. of patients (n)	17	20	
Male/female (n)	06/11	10/10	
Mean age (years)	40.71 ± 17.04	59.18 ± 13.71	< 0.0001
Hospital stay (days)	23.50 ± 9.54	23.10 ± 10.04	0.9147
Surgery time (min)	177.39 ± 39.29	231.70 ± 65.31	< 0.0001
Hospitalization cost	74,881 ± 34,374	78,339 ± 25,327	< 0.0001
Blood loss (ml)	400.00 ± 357.3	532.40 ± 303.60	< 0.0001
Drainage time (days)	6.21 ± 0.90	7.12. ± 0.85	0.2985
Mean fusion time (months)	11.30 ± 4.75	6.80 ± 1.50	< 0.0001
Fever	12/17	12/20	0.9441
Antibiotic therapy time before surgery (days)	17.62 ± 3.76	13.86 ± 4.71	0.4328
Affected levels			
L1–2	4	3	
L2–3	1	2	
L3–4	1	2	
L4–5	8	9	
L5–S1	3	4

**Table 2:** Clinical and radiographic outcomes

Parameter	Group A	Group B	P value
ESR			
Before treatment	56.43 ± 37.47	64.76 ± 33.47	0.5183
Final FU	14.93 ± 3.79	15.24 ± 3.15	0.8074
CRP			
Before treatment	34.25 ± 31.23	34.49 ± 26.14	0.8441
Final FU	5.07 ± 0.75	5.82 ± 0.38	0.0619
VAS			
Before treatment	6.95 ± 0.94	6.5 ± 0.75	0.8023
Final FU	1.95 ± 0.69	1.58 ± 0.95	0.9607
ODI			
Before treatment	40.95 ± 4.10	41.05 ± 4.25	0.8901
Final FU	5.10 ± 1.50	5.60 ± 1.85	0.6675
Segmental angle (°)			
Before treatment	15.85 ± 3.60	14.75 ± 4.15	0.1955
Final FU	10.25 ± 2.05	9.08 ± 3.45	0.098
Intervertebral height (cm)			
Before treatment	10.30 ± 2.80	11.50 ± 2.10	0.1065
Final FU	9.50 ± 1.05	10.10 ± 1.30	0.542

ODI: Oswestry Disability Index; VAS: visual analogue scale; ESR: erythrocyte sedimentation rate; CRP: C-reactive protein; FU: follow-up

**Table 3:** Serological and bacteriological findings

Parameter	Group A	Group B	P value
Blood culture	6/17	4/20	0.3098
*Aerobacter cloacae*	1		
*Staphylococcus aureus*	2	1	
*Escherichia coli*	2		
*Streptococcus viridis*	1		
*Staphylococcus warneri*		1	
*Klebsiella pneumoniae*		2	
Pathological findings	17/17	20/20	0.9899

### Radiological assessments

**Fig. 3:**
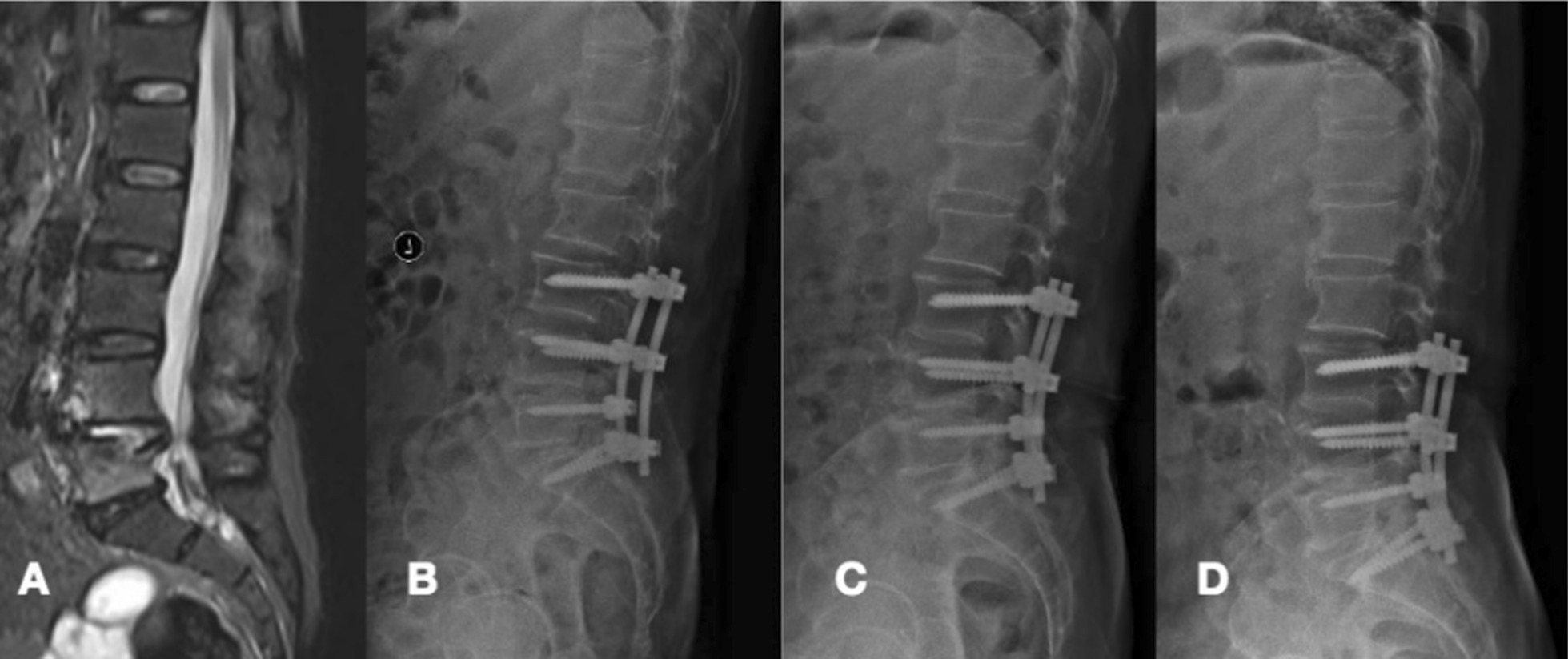
A patient aged 40–50 years old with lumbar pyogenic discitis (L4–5) underwent posterior lumbar interbody fusion combined with instrumentation with instrumentation with an IG. **A** Preoperative computed tomography (MRI) showed bone destruction of the L4–5 disc and compression of the spinal nerves. **B**, **C** Six-month and 12-month postoperative X-rays showed maintained correction. **D** Three-year postoperative X-rays showed that solid bone fusion had been achieved

**Fig. 4:**
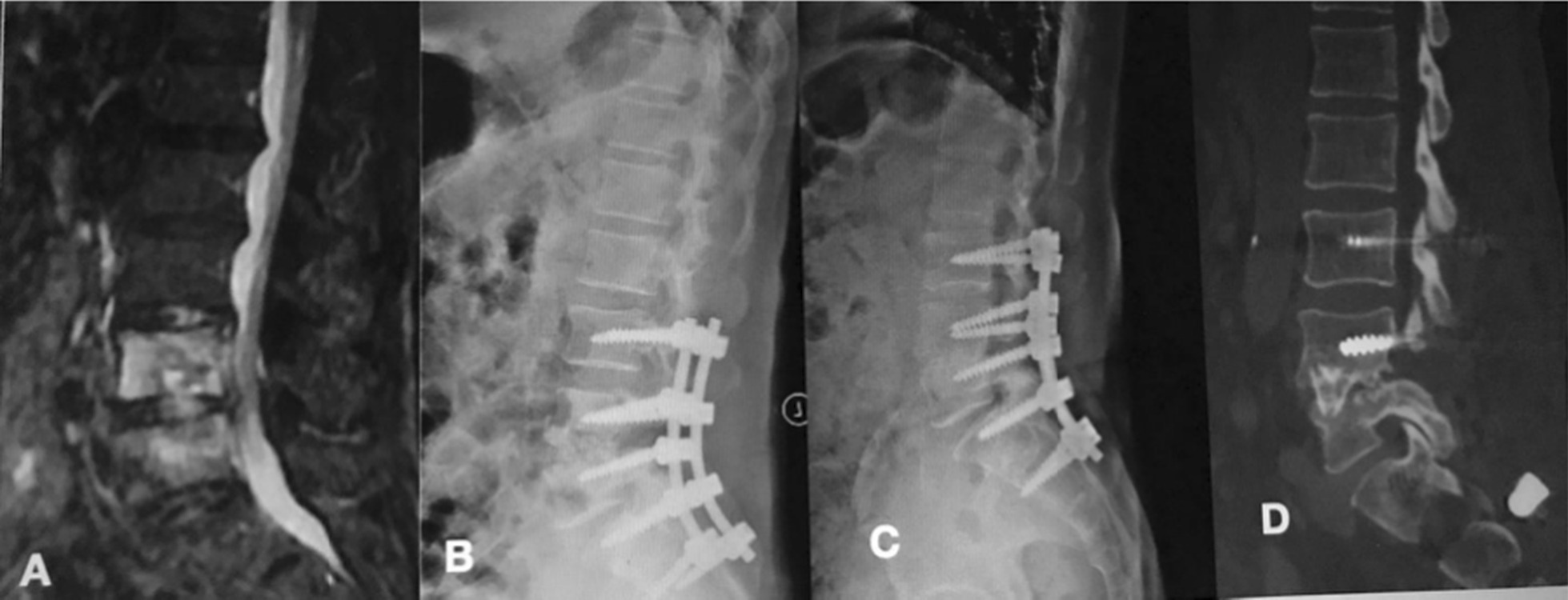
A patient aged 30–40 years old with lumbar pyogenic discitis (L4–5) underwent single-segment posterior lumbar interbody fusion combined with instrumentation with an LSP. **A** Pretreatment MRI showed the destruction of the L4–5 disc and concomitant compression of the spinal nerves. **B** Twelve-month postoperative X-rays showed maintained correction. **C**, **D** At the 32-month FU, plain X-ray and CT showed the good correction and reliable fusion

LPS was fully cured, and the bone fusion at a mean time of 11.30 ± 4.751 months in group A was longer than that in group B (6.80 ± 1.50) (Figs. 3, 4). There were no significant differences in the mean segmental angle or mean interventricular height preoperation and at the final FU (P > 0.05).

### Complications

Some postoperative complications occurred, such as superficial infection (4 cases in group A and 5 in group B), which healed with dressing changes.

## Discussion

LPS, as one of the ancient diseases, is a rare infection which had a hidden onset, slow course. With the increase of HIV Co infection, bacterial drug resistance and population migration [1–5]. LPS diagnosis can be very difficult, especially in a resource-poor environment. The diagnosis of LPS is usually delayed for months and may be misdiagnosed as DDD [5–9]. This challenges stem from the relative rarity of the disease, the much higher incidence of non-specific lower back pain in the population, changes in protein expression, non-pathogenic imaging and positive rates from culture [10, 11]. The main means of diagnosis are spine imaging and spine biopsy materials for microbiological examination and ideal histopathology. In any infectious disease, the therapy should be based on the correct results of culture and in vitro chemosensitivity assay. The most common bacterium of LPS is *Staphylococcus aureus*. According to Infectious Diseases Society of America (IDSA) clinical practice guideline for the diagnosis and treatment of native vertebral osteomyelitis in adults, LPS is frequently monomicrobial and most often due to *Staphylococcus aureus *[5, 6, 12, 13]. The local administration of VCM was usually performed for better control of infection. However, in our study, although blood culture isolated the bacterium, the positive culture rate was 35.2% in group A and 20% in group B, but there were no findings in the stool, urine or surgical material culture [14, 15]. There were only 3 cases of *Staphylococcus aureus* in the two groups. Perhaps because the regimens of antibiotic therapy and the methods of administration were empirical, in the study, the mean times of antibiotic therapy before surgery, which were 17.62 ± 3.76 days in group A and 13.86 ± 4.71 days in group B, could explain why we had more difficulty with the diagnosis as well as the morbidity and mortality of effective treatment, and the culture positive rate was low.

Most patients are treated with a course of 6-week or more of antibiotic therapy, but a few cases maybe need surgical debridement and/or spinal reconstruction during or after antibacterial treatment. After complete lesion debridement, numerous interbody bone grafts are applied to recover and reconstruct spinal stability [16–18]. Titanium mesh cages (TMCs) filled with autologous bone grafts have been widely applied and could achieve high bony fusion rates. However, the surgical planning and results could be affected by the subsidence, stress shielding, and radio-opacity [19]. Thus, our research aimed to find a new bone graft that could provide biomechanical support and achieve bony fusion to reduce the incidence of complications. The use of a LSP has several strengths. Firstly, compared with the IG, the LSP were more minimally invasive, shorten the surgery time, and reduce postoperative complication rates. Secondly, in the study, the mean time of bone fusion was 11.30 ± 4.751 months in group A, which was longer than that in group B (6.80 ± 1.50). Although the LSP gained a longer time of bone healing, with the correction of segmental kyphosis, there was no significance among the groups. Hence, the LSP could provide excellent biomechanical support, bone fusion properties and maintain good alignment. Furthermore, the LSP, as an autologous bone graft, has a cortical bony structure supporting the bone defect space and can ensure and maintain segmental stability and alignment. After the surgery, the VAS and ODI scores were improved significantly, which improved the life quality of the patients. In the absence of sources of autogenous or allogeneic bone, the LSP could be good choice for the patients, which could achieved satisfactory clinical results and could shorten hospital stay and cost.

Although the diagnosis of LPS is very difficult, some clues can be identified: severe low back pain, fever, increasing infection indexes, magnetic resonance imaging (MRI), C-arm-guided biopsy, and clear pathogenic bacteria from cultures [16, 17, 20–22]. However, there is a50% misdiagnosis rate, and pathological findings are still the gold standard despite the culture of blood, urine, stool, or surgical tissue being negative. MRI is considered the modality of choice for radiographic diagnosis, especially in severe lower back pain. Previous studies have reported an MRI sensitivity of 96%, a specificity of 93% and an accuracy of 94% in LPS, and MRI plays the key role in the continuous observation of LPS [23–26].

However, we declare that the study had a few limitations. First, the retrospective nature of our study was associated with bias, more patients need to be included in the study. Second, the single LSP as a bone graft had a long bony fusion time and may be a risk factor for the delay of bony fusion. Third, the study did not consider intra- or inter-observer differences, which was related to bias. Thirdly, although the strengths and weaknesses of the therapy plans were completely explained before the surgery, there was selection bias. The prospective, randomized studies with long-term FU will be performed in the future.

## Conclusion

Our study results showed that the use of a LSP could be a new effective procedure in treating single-segment LPS in carefully selected patients, resulting in good bone fusion and spinal stability restoration, as it could be a reliable and effective bone grafting method.

## Data Availability

The datasets used and/or analyzed during the current study are available from the corresponding author on reasonable request.

## Abbreviations

LSP: Spinous process
IG: Iliac graft
VAS: Visual analog scale
ODI: Oswestry Disability Index
ESR: Erythrocyte sedimentation rate
CRP: C-reactive protein
FU: Follow-up
LPS: Lumbar pyogenic spondylodiscitis
HIV: Human immunodeficiency virus
VCM: Vancomycin
